# Imagem Cardiovascular em Pacientes com COVID-19

**DOI:** 10.36660/abc.20201356

**Published:** 2021-08-09

**Authors:** Rujittika Mungmungpuntitpantip, Viroj Wiwanitkit

**Affiliations:** 1 Dr. DY Patil University Pune Índia Dr. DY Patil University, Pune – Índia

**Keywords:** Doenças Cardiovasculares, Pneumopatias, Coronavírus, COVID-19, Pandemia, Diagnóstico por Imagem, Pacientes Assimtomáticos

Caro Editor,

Gostaríamos de compartilhar ideias relacionadas à publicação “Imagem Cardiovascular em Pacientes com COVID-19.”^1^ Grossman e Lima concluíram que “cardiologistas nucleares e médicos nucleares devem estar cientes dos achados incidentais em pacientes assintomáticos com COVID-19 e devem otimizar os protocolos de CPM, quando o procedimento for necessário.”^1^ Os resultados desse estudo estão de acordo com um relato anterior da Ásia.^2^ A imagem pode ajudar a identificar problemas cardíacos e pulmonares que podem ocorrer de modo assintomático devido à COVID-19 ou a uma patologia silenciosa anterior.^2^ Um ponto importante é o diagnóstico diferencial da lesão nova e da patologia anterior subjacente. Nos países tropicais, pode existir uma patologia comum, como a tuberculose, que resulta em dificuldades de interpretação de novos problemas cardíacos e pulmonares devido à COVID-19.^3^ Considerando-se que a interpretação das imagens depende principalmente do radiologista, torna-se necessário que os radiologistas tenham maior consciência e cuidado ao interpretar as imagens clínicas durante a pandemia de COVID-19.
