# Supplementary material for: Imagem Cardiovascular em Pacientes com COVID-19
Source: Arq Bras Cardiol. 2021 Aug 9;117(2):415. [Article in Portuguese] doi: 10.36660/abc.20201356 (PMC8395790; doi:10.36660/abc.20201356)
Supplement: Translation

# Translation: Cardiovascular Imaging in COVID-19

**Authors:** Rujittika Mungmungpuntitpantip, Viroj Wiwanitkit

**Affiliations:** 1 Pune India Dr. DY Patil University, Pune – India

**Keywords:** Cardiovascular Diseases, Lung Diseases, Coronavirus, COVID-19, Pandemics, Diagnostic Imaging, Asymptomatic Patients

Dear Editor,

We would like to share ideas on the publication “Cardiovascular Imaging in Patients with COVID-19.”^1^ Grossman and Lima concluded that “nuclear cardiologists and nuclear medicine physicians must be aware of incidental findings in asymptomatic patients with COVID-19, and they should optimize MPI protocols, when the procedure is necessary.”^1^ The results in this study are concordant with a previous report from Asia.^2^ Imaging can help identify both heart and lung problems that might asymptomatically occur due to COVID-19 or a previous silent pathology.^2^ An important point is the differential diagnosis of the new lesion and the previous underlying pathology. In tropical countries, there might be a common pathology, such as tuberculosis, that results in difficulty in interpreting new heart and lung problems due to COVID-19.^3^ Since image interpretation depends mainly on the radiologist, it is necessary for radiologists to increase awareness and concern when interpreting clinical images during the COVID-19 pandemic.
